# Leucine-rich repeat kinase-1 regulates osteoclast function by modulating RAC1/Cdc42 Small GTPase phosphorylation and activation

**DOI:** 10.1152/ajpendo.00189.2016

**Published:** 2016-09-06

**Authors:** Canjun Zeng, Helen Goodluck, Xuezhong Qin, Bo Liu, Subburaman Mohan, Weirong Xing

**Affiliations:** ^1^Musculoskeletal Disease Center, Jerry L. Pettis Memorial Veterans Affairs Medical Center, Loma Linda, California;; ^2^Department of Medicine, Loma Linda University, Loma Linda, California;; ^3^Department of Orthopedics, The Third Affiliated Hospital Of Southern Medical University, Guangzhou, China;; ^4^Department of Orthopedics, The Third Xiangya Hosptial, Central South University, Changsha, Hunan, China

**Keywords:** Lrrk1, bone resorption, osteoclast, RAC1, Cdc42, protein kinase, protein phosphorylation

## Abstract

Leucine-rich repeat kinase-1 (Lrrk1) consists of ankyrin repeats (ANK), leucine-rich repeats (LRR), a GTPase-like domain of Roc (ROC), a COR domain, a serine/threonine kinase domain (KD), and WD40 repeats (WD40). Previous studies have revealed that knockout (KO) of *Lrrk1* in mice causes severe osteopetrosis, and a human mutation of Lrrk1 leads to osteosclerotic metaphysial dysplasia. The molecular mechanism by which Lrrk1 regulates osteoclast function is unknown. In this study, we generated a series of Lrrk1 mutants and evaluated their ability to rescue defective bone resorption in Lrrk1-deficient osteoclasts by use of pit formation assays. Overexpression of Lrrk1 or LRR-truncated Lrrk1, but not ANK-truncated Lrrk1, WD40-truncated Lrrk1, Lrrk1-KD, or K651A mutant Lrrk1, rescued bone resorption function of Lrrk1 KO osteoclasts. We next examined whether RAC1/Cdc42 small GTPases are direct substrates of Lrrk1 in osteoclasts. Western blot and pull-down assays revealed that Lrrk1 deficiency in osteoclasts resulted in reduced phosphorylation and activation of RAC1/Cdc42. In vitro kinase assays confirmed that recombinant Lrrk1 phosphorylated RAC1-GST protein, and immunoprecipitation showed that the interaction of Lrrk1 with RAC1 occurred within 10 min after RANKL treatment. Overexpression of constitutively active Q61L RAC1 partially rescued the resorptive function of Lrrk1-deficient osteoclasts. Furthermore, lack of Lrrk1 in osteoclasts led to reduced autophosphorylation of p21 protein-activated kinase-1 at Ser^144^, catalyzed by RAC1/Cdc42 binding and activation. Our data indicate that Lrrk1 regulates osteoclast function by directly modulating phosphorylation and activation of small GTPase RAC1/Cdc42 and that its function depends on ANK, ROC, WD40, and kinase domains.

there are two major known causes of osteoporosis: low peak bone mineral density (BMD), typically achieved around the age of 30, and a high bone loss rate occurring particularly after menopause and during the natural process of aging. Bone loss occurs with age in part because the rate of bone resorption surpasses the rate of bone formation. Effective inhibitors of bone resorption such as bisphosphonates and an anti-RANKL antibody have now been used clinically to treat bone diseases. Both of these antiresorptive agents act via inhibition of both osteoclast formation and activity, but treatment with these bone resorption inhibitors results in suppression of bone formation in addition to bone resorption. Long-term treatment with these drugs may be associated with impaired fracture healing, jaw osteonecrosis, and increased risk for atypical fractures of the thigh (2, 18, 24, 26, 28, 31). Bisphosphonate toxicity during childhood can impair skeletal modeling and remodeling, with structural changes that evolve and persist into adult life (32). Therefore, there is a need for identification of novel alternative antiresorptive drug targets for osteoporosis treatment that avoid the antianabolic actions of currently available drugs.

In our previous studies, we focused on understanding the role of a novel gene, leucine-rich repeat kinase-1 (*Lrrk1)*, in regulation of osteoclast function, and we showed that disruption of Lrrk1 function resulted in the highest observed BMD phenotype among 3,629 distinct knockout (KO) mouse lines tested (36). Mice with disruption of Lrrk1 had impaired osteoclast activity but not osteoclast formation. While bone resorption was reduced dramatically, bone formation was not affected in the *Lrrk1* KO mice. The increased BMD in *Lrrk1* KO mice was higher than in *Sost* and *c-Src* KO mouse lines (36). In aging *Lrrk1* KO mice, BMD of the long bones and spine still remained elevated. In addition, *Lrrk1* KO mice responded normally to PTH anabolic treatments but were resistant to ovariectomy-induced bone loss. Consistent with our observations in the *Lrrk1* KO mouse model, our recent human genetic studies have identified a severe osteopetrosis phenotype at the vertebra and the metaphysis of long and short tubular bones in a patient with *Lrrk1* deletion and frame-shifted mutation (15). These observations make Lrrk1 an attractive antiresorptive drug target because Lrrk1 KO mice have normal membranous bone, and inhibiting Lrrk1 may not interfere with osteoclast-coupled bone formation in the maxilla and mandible or cause osteonecrosis of the jaw. In terms of signaling pathways by which Lrrk1 regulates osteoclast functions, our previous work has shown that Tyr^527^ phosphorylation of c-Src was altered in Lrrk1-deficient osteoclasts. It is known that c-Src is inactivated by several phosphatases and two kinases, Csk and Csk homologous kinase (Chk), both of which phosphorylate Tyr^527^ of c-Src. Thus, Csk and Chk could be potential candidate substrates of Lrrk1 in osteoclasts. Kedashiro and colleagues recently reported that Lrrk1 regulates EGFR trafficking by phosphorylating CLIP-170, promoting association of CLIP-170 with dynein-dynactin complex formation, and recruiting p150^Glued^ to microtubule plus ends in HEK-293 human kidney cells (12, 17). More recently, Barrera et al. reported that Lrrk1 phosphorylates DCK5RAP2 in its γ-tubulin-binding motif to promote the interaction of CDK5RAP2 with γ-tubulin. Lrrk1 phosphorylation of CDK5RAP2 at Ser^140^ is necessary for mitotic spindle orientation (13). Though CLIP-170 is involved in cytoskeleton arrangement, male mice with disruption of CLIP-170 exhibited abnormal sperm and reduced fertility without skeletal phenotypes (1), whereas Lrrk1-deficient males showed normal fertility but severe osteopetrosis (36). Mice with loss of CDK5RAP2 function exhibited small size, kyphosis, severe anemia, and neonatal death, which are in contrast to *Lrrk1* KO mice (3, 22). These studies indicate that neither CLIP-170 nor CDK5RAP2 is likely a biological substrate of Lrrk1 in osteoclasts. Therefore, the direct substrates of Lrrk1 in osteoclasts need to be determined.

In this study, we performed a series of Lrrk1 deletion and functional studies and examined whether RAC1/Cdc42 small GTPase proteins are direct substrates of Lrrk1 in osteoclasts. Our studies suggest that *1*) the ROC, ANK, and WD40 domains of Lrrk1 are critical for Lrrk1 function, and *2*) Lrrk1 may regulate osteoclast function via modulation of RAC1/Cdc42 serine/threonine phosphorylation and activation; as such RAC1/Cdc42 may be key biological substrates of Lrrk1 in osteoclasts.

## MATERIALS AND METHODS

### 

#### Recombinant proteins, antibodies, and plasmids.

Recombinant macrophage colony-stimulating factor (M-CSF) and RANKL and monoclonal anti-histidine antibody were from R&D Systems (Minneapolis, MN). Anti-Rac1/Cdc42, anti-pS71-RAC1/Cdc42, anti-pSer^473^-Akt, anti-Akt, anti-PAK1/2/3, anti-pSer^144^-PAK1, anti-pS21-PAK1, anti-pThr^423^-PAK1, anti-PKC substrate serine/threonine motif, and anti-ATM/ATR substrate serine/threonine motif antibodies were products of Cell Signaling Technology (Danvers, MA). Monoclonal antibodies against Flag (M5) and β-actin and anti-M2-conjugated agarose resin were purchased from Sigma (St. Louis, MO). Recombinant human RAC1-GST, Ni-NTA His tag purification kit, and anti-mouse IgG magnetic beads were obtained from Life Technologies (Carlsbad, CA). Rac1 activation assay kit was purchased from Cell Biolabs, (San Diego, CA). Plasmids of pUC57 encoding human Lrrk1 (hLrrk1), mouse Lrrk1 (mLrrk1), ANK truncated mLrrk1 (ΔANK-mLrrk1), LRR truncated mLrrk1 (ΔLRR-mLrrk1), WD40 truncated mLrrk1 (ΔWD-mLrrk1), mLrrk1 kinase domain (mLrrk1-KD), and K651A mutant mLrrk1 (K651A-mLrrk1) were synthesized by GenScript (Piscataway, NJ) and subcloned into the pRRLsin-cPPT-SFFV-GFP-wpre plasmid (Addgene, MA) by replacing GFP with Lrrk1 coding DNA fragments. All constructs contain 3xFlag tags at the NH_2_ terminus and 6xhis tags at the COOH terminus.

#### Generation of DNA replication-deficient recombinant virus and viral transduction.

Lentiviral particles were generated by cotransfection of pRRLsin-cPPT-SFFV-Lrrk1-wpre plasmid or Lrrk1 mutant plasmid with Pax2 and pMD2.G constructs in 293T cells, and transduction of primary osteoclast precursors was carried out as described previously (36). Constitutively active recombinant adenovirus of RAC L61 (Q61L) was purchased from Cell Biolabs. Adenovirus GFP (Ad-GFP) was generated in Ad-293 cells as described previously (7). To transduce the primary osteoclast precursors, monocytes isolated from the spleen or bone marrow were first induced to proliferate in the presence of M-CSF (20 ng/ml). After 3 days of expansion, the proliferating cells were trypsinized and seeded on bone slices in 48-well plates at a density of 10,000 cells/well. The cells were incubated with M-CSF overnight and transduced by adding lentiviral particles at 5 multiplicity of infection (MOI) or adenovirus at 20 MOI in the presence of 8 μg/ml polybrene and 20 ng/ml M-CSF for 24 h followed by replacement of a fresh osteoclast differentiation medium containing 20 ng/ml M-CSF and 30 ng/ml RANKL.

#### Mice.

*Lrrk1*-deficient (*Lrrk1*^*−/−*^) mice were generated via targeted disruption of coding exons 16–19 of the GTPase domain of the *Lrrk1* gene, as described previously (36). Mice were housed at the VA Loma Linda Healthcare System (VALLHCS) under standard approved laboratory conditions. All procedures were performed with approval of the Institutional Animal Care and Use Committee of VALLHCS.

#### In vitro osteoclast formation and bone resorption pit assays.

Primary osteoclast precursors were isolated from the spleen or bone marrow of the femurs and tibias from 5-wk-old mice as described previously (36). The isolated precursors were maintained in α-MEM supplemented with 10% fetal bovine serum, penicillin (100 U/ml), streptomycin (100 μg/ml), and M-CSF (20 ng/ml) at 37°C in 5% CO_2_ for 3 days to stimulate monocyte proliferation. To induce osteoclast differentiation, trypsinized precursors were seeded in 48-well plates (10,000 cells/well) and incubated with M-CSF (20 ng/ml) and RANKL (30 ng/ml). The medium was changed every 2 days. Osteoclastogenesis was evaluated by counting TRAP staining positive, multinucleated cells having at least three nuclei. For bone resorption pit assays, slices from bovine cortical bone were placed in 48-well plates, and cells were differentiated on top of the bone slices as described above. Cells on bone slices were digested with trypsin at 37°C overnight. Multinucleated cells were further removed by 5-min sonication in 1 M ammonia. Bone slices were washed with PBS and stained with hematoxylin for 5 min, followed by three washes with PBS. The entire surface of each bone slice was examined, and the total resorbed area per bone slice was quantified using ImageJ software (National Institutes of Health).

#### Recombinant mLrrk1 purification and in vitro kinase assay.

293T cells expressing mLrrk1 were lysed in lysis buffer (50 mM Tris·HCl, pH 8.0, 150 mM NaCl, 1% NP-40, 1× protease inhibitor cocktail, and 1× phosphatase inhibitor cocktail) for 20 min on ice followed by centrifugation at 12,000 rpm for 10 min 4°C. The lysate was then incubated with Ni-NTA resin overnight at 4°C for purification of polyhistidine-mLrrk1 protein with the HisPur Ni-NTA Spin Purification Kit according to manufacturer's instructions. The Lrrk1 kinase assay was performed as described previously (20). Briefly, the purified mLrrk1 was preincubated in a kinase buffer containing 10 μM GTPγS on ice for 20 min. Kinase reactions were carried out in a final volume of 25 μl containing 1 μg GTPγS-bound mLrrk1 protein, 50 mM Tris·HCl (pH7.5), 5 mM MgCl_2_, 5 mM MnCl_2_, 0.5 mM DTT, 1× phosphatase inhibitor cocktail, 0.5 μg recombinant GST-RAC1, 3 μM ATP, and 5 μCi of [γ-^32^P]ATP. Samples were incubated for 45 min at 30°C. Reactions were terminated by the addition of LDS sample buffer and heated at 90°C for 10 min. GST-RAC1 was resolved by SDS-NuPAGE (Life Technologies). Gels were dried, and phosphorylation of GST-RAC1 was detected by autoradiography.

#### RAC1 activation assay.

Primary osteoclast precursors derived from the spleen of *Lrrk1* KO and WT control mice were differentiated into multinuclear cells as described previously (36). Mature osteoclasts were cultured in RANKL-free medium for 4 h and then treated with 30 ng/ml RANKL for 30 min prior to cellular protein extraction. The Rac1 activation assay was carried out using Cell Biolabs's Rac1 Activation Assay Kit according to the manufacturer's instructions. Briefly, the active form of Rac1 was selectively pulled down from the lysate of differentiated osteoclasts with the p21-binding domain (PBD) of p21 protein-activated kinase (PAK) agarose beads. Subsequently, the precipitated GTP-Rac1 was detected by Western blot analyses using specific anti-Rac1 monoclonal antibody.

#### Immunoprecipitation.

RAW 264.7 cells were transduced with lentivirus expressing hLrrk1 as described above. Cells were differentiated in α-MEM containing 10% FBS, 30 ng/ml RANKL and 20 ng/ml M-CSF for 4 days and then cultured in RANKL- and serum-free medium for 4 h followed by RANKL restimulation. Subsequently, the cells were lysed with lysis buffer and precleared with protein A/G-Sepharose prior to immunoprecipitation with anti-Flag or control IgG. Interactions of Lrrk1 with RAC1 were detected by Western blot using anti-Rac1 antibody (35).

#### Western blot analyses.

Differentiated osteoclasts were cultured in serum- and RANKL-free medium for 4 h and then treated with RANKL for 30 min before harvesting. The cells were lysed, and cellular lysates were analyzed by Western blot using antibodies specific to the ATM/ATR substrate and PKC substrates, Flag, phosphor-RAC1/Cdc42, phosphor-PAK1, phosphor-Akt, and β-actin, as described (37).

#### Statistical analyses.

Data are expressed as means ± SE and were analyzed using Student's *t*-test or two-way ANOVA. Values were considered statistically significant when *P* < 0.05.

## RESULTS

### 

#### ANK, ROC, and WD-40 domains of Lrrk1 are essential for bone resorptive function.

**Fig. 1.**
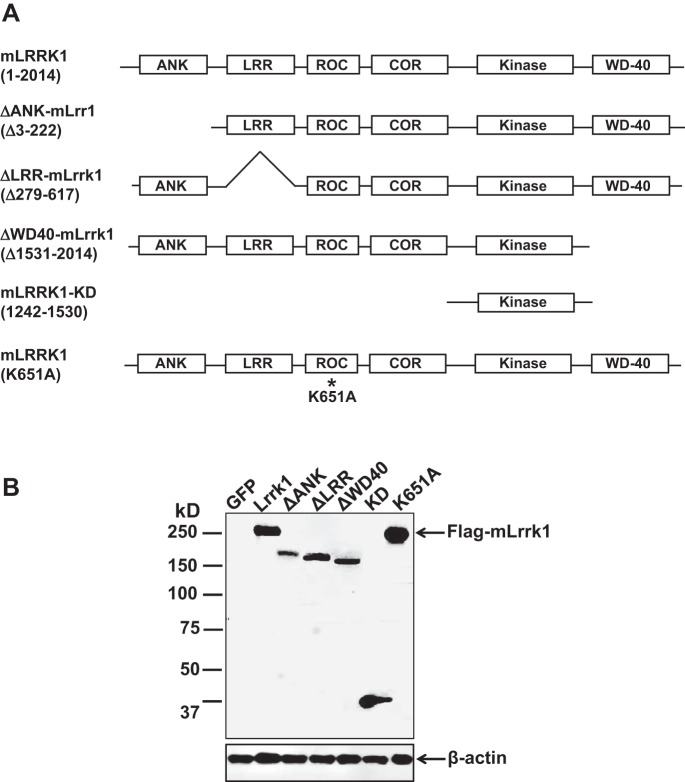
Lentivirus-mediated expression of mouse leucine-rich repeat kinase-1 (mLrrk1), truncated mLrrk1, and kinase-dead mutant mLrrk1. *A*: schematic diagrams of Lrrk1 deletions and mutation. *B*: lentivirus-infected 293T cells express the expected sizes of mLrrk1, ΔANK-mLrrk1, ΔLRR-mLrrk1, ΔWD-mLrrk1, mLrrk1-KD, and K651A-mLrrk1 proteins. An aliquot of 30 μg total cellular protein was separated on LDS-NuPage and analyzed by Western blot with specific antibody against Flag tag at the NH_2_ terminus of the fusion proteins.

To study the structure and function of Lrrk1, we generated the complete coding sequence of mLrrk1, ΔANK-mLrrk1, ΔLRR-mLrrk1, ΔWD-40-mLrrk1, mLrrk1-KD, and K651A-mLrrk1, as shown in Fig. 1*A*. To validate the constructs and test the expression levels of the Flag-tagged Lrrk1 proteins, 293T cells were transduced with lentiviral particles at a MOI of 5, and total cellular proteins were analyzed by Western blot with an antibody against Flag tag. As shown in Fig. 1*B*, the infected 293T cells expressed high levels of the predicted sizes of full-length mLrrk1, ΔANK-mLrrk1, ΔLRR-mLrrk1, ΔWD-mLrrk1, mLrrk1-KD, and K651A-mLrrk1.

**Fig. 2.**
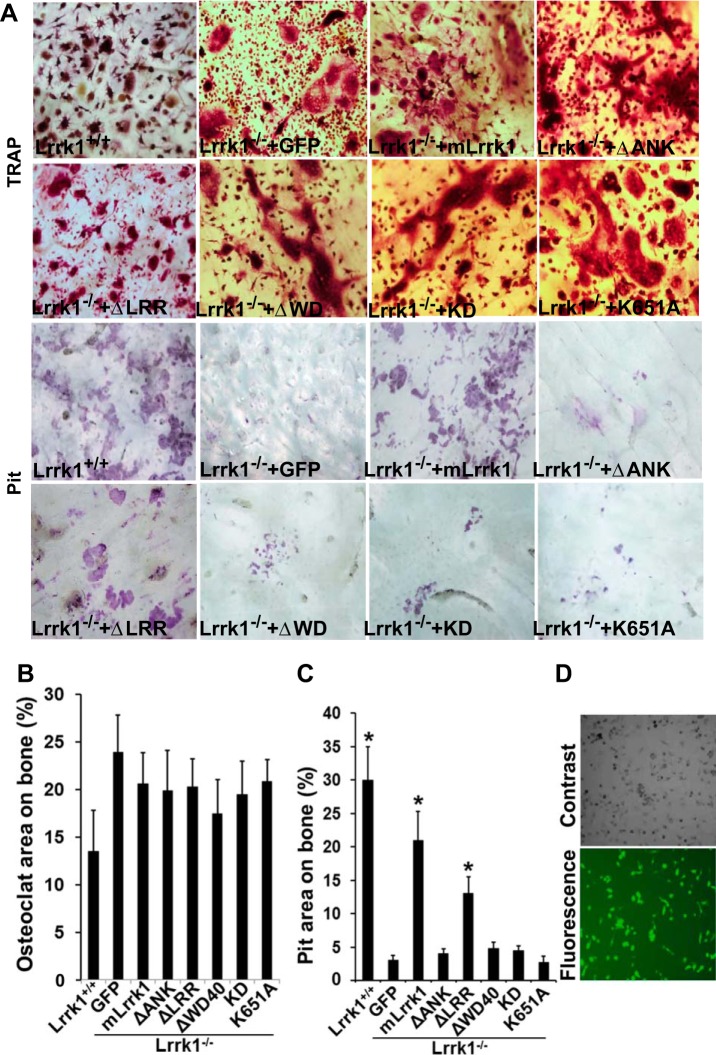
ANK, ROC, and WD-40 domains of Lrrk1 are essential for bone resorptive function. *A*: representative images of TRAP staining-positive osteoclasts and resorptive pits on bone slices. *B* and *C*: quantitative data of multinucleated cells and pit area on bone slices. *Statistical significance vs. osteoclasts expressing GFP (*P* < 0.01, *n* = 6). *D*: lentivirus-mediated GFP expression in primary osteoclast precursors.

To identify the functional domain of Lrrk1 in osteoclasts, Lrrk1-null monocytes derived from Lrrk1 KO mice were transduced with lentiviral constructs that express control GFP, mLrrk1, ΔANK-mLrrk1, ΔLRR-mLrrk1, ΔWD-mLrrk1, mLrrk1-KD, or the mutant K651A-mLrrk1 that abolishes GTP binding and inactivates Lrrk1 kinase activity (kinase-dead Lrrk1) (20, 21). We used Lrrk1-deficient osteoclast precursors to prevent interference from endogenous Lrrk1 in the WT monocytes. The infected cells were then induced to form mature osteoclasts on bone slices for TRAP staining and bone resorptive pit assays. As shown in the Fig. 2*A*, lentivirus infection of osteoclast precursor cells expressing GFP, mLrrk1, or truncated mLrrk1 protein did not affect osteoclast formation on bone slices when the same number of precursors (10,000 cells/well of 48-well plate) was seeded and cultured under identical culture conditions. There were no differences in the numbers of TRAP-positive osteoclasts generated from M-CSF- and RANKL-treated bone marrow macrophages derived from WT and Lrrk1 KO mice in plastic tissue culture dishes (data not shown) (36). However, Lrrk1-deficient osteoclasts behaved differently on bone slices compared with WT Lrrk1 osteoclasts. Dysfunctional Lrrk1-deficient cells on the bone surface failed to resorb bone but continued to fuse together to become huge multinuclear cells with more than 100 nuclei, whereas the WT osteoclasts were much smaller but were active in resorbing bone. The size of dysfunctional osteoclasts lacking Lrrk1 was five times larger than the WT cells (1 ± 0.1 vs. 5 ± 2.3, *P* < 0.05). The number of the nuclei in Lrrk1-deficient mature osteoclasts was significantly greater than in the WT multinucleated cells (28 ± 9 vs. 113 ± 60, *P* < 0.05). Consistent with the in vitro osteoclast differentiation assays on bone slices, similarly enlarged abnormal osteoclasts were also observed on the bone surface of Lrrk1 KO mice by histological analysis (36). Lentivirus-mediated expression of mLrrk1, ΔANK-mLrrk1, ΔLRR-mLrrk1, ΔWD-mLrrk1, mLrrk1 KD, or a K651A kinase-dead mLrrk1 in Lrrk1-deficient precursors had no effect on osteoclast differentiation on bone surfaces (Fig. 2*A*). The areas of mature TRAP-positive osteoclasts on bone slices were comparable for *Lrrk1*-deficient osteoclasts expressing GFP, mLRRK1, and different Lrrk1 mutants (Fig. 2*B*). Whereas expression of mLrrk1 or ΔLRR-mLrrk1 in Lrrk1-deficient cells rescued defective bone-resorptive function of mature osteoclasts, overexpression of control GFP, ΔANK-mLrrk1, ΔWD-mLrrk1, mLrrk1-KD, or K651A mutant mLrrk1 did not rescue defective osteoclast function (Fig. 2, *A, B, C*). The area of resorption pits was reduced by 90% in *Lrrk1*-deficient GFP-expressing cells compared with that of the WT cells (Fig. 2*C*). There were no significant changes in pit areas in the cells expressing ΔANK-mLrrk1, ΔWD-mLrrk1, mLrrk1-KD, or K651A mutant mLRRK1 compared with those cells expressing GFP. The individual resorption pits remained smaller and shallower in osteoclast cultures expressing the ΔANK-mLrrk1, ΔWD-mLrrk1, mLrrk1-KD, or K651A mutant mLRRK1. It should be noted that the expression of mLrrk1 or ΔLRR-mLrrk1 did not completely rescue bone-resorptive function of Lrrk1-null osteoclasts because the transduction efficiency of lentiviral particles in primary cultures of monocytes derived from mouse spleen and bone marrow was ∼70%, as measured by counting GFP expressing cells 24 h after lentiviral transduction (Fig. 2*D*).

#### Lrrk1 phosphorylates and activates RAC1/Cdc42 small GTPase proteins in osteoclasts.

**Fig. 3.**
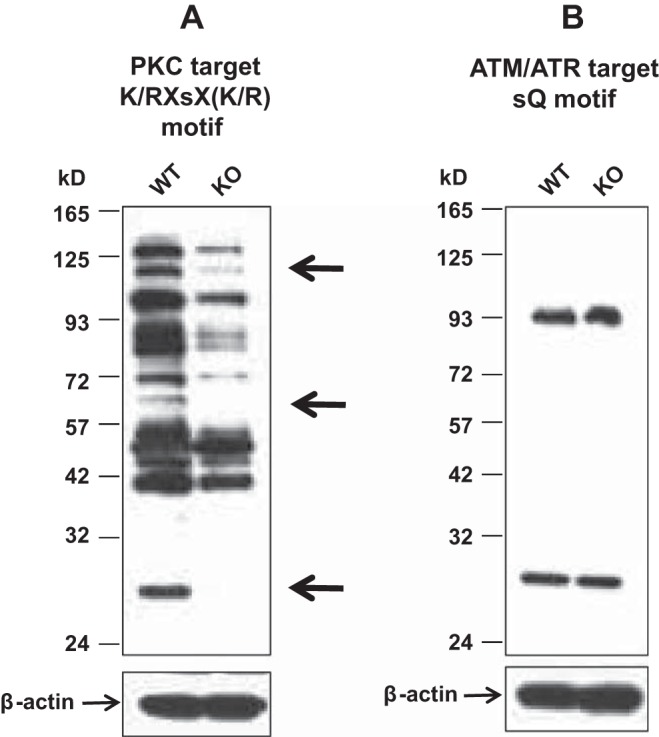
Reduced levels of serine/threonine phosphorylated proteins in Lrrk1 KO osteoclasts. Osteoclast precursors derived from WT and Lrrk1 KO mice were differentiated in the presence of RANKL and macrophage colony-stimulating factor (M-CSF) for 4 days, and cells were cultured in serum- and RANKL-free medium for 4 h followed by RANKL stimulation for 30 min. Cellular lysates were analyzed by Western blot using anti-phospho-serine/threonine motif antibodies specific to the PKC substrates A and ATM/ATR substrate B, respectively. Expression of β-actin was used as a loading control. Arrows indicate reduced phosphorylated species of cellular proteins in Lrrk1-deficient osteoclasts.

To determine whether protein phosphorylation is influenced by a lack of Lrrk1 in osteoclasts, Western blot analysis was carried out with anti-phospho-serine/threonine motif antibodies using osteoclast lysates from WT and KO mature osteoclasts that were treated with RANKL for 30 min. We found that PKC substrates with molecular masses of ∼110, 55–78, and 27 kDa were phosphorylated at significantly lower levels in *Lrrk1* KO osteoclasts compared with WT cells, whereas phosphorylation of ATM/ATR substrates was unchanged (Fig. 3, *A* and *B*).

**Fig. 4.**
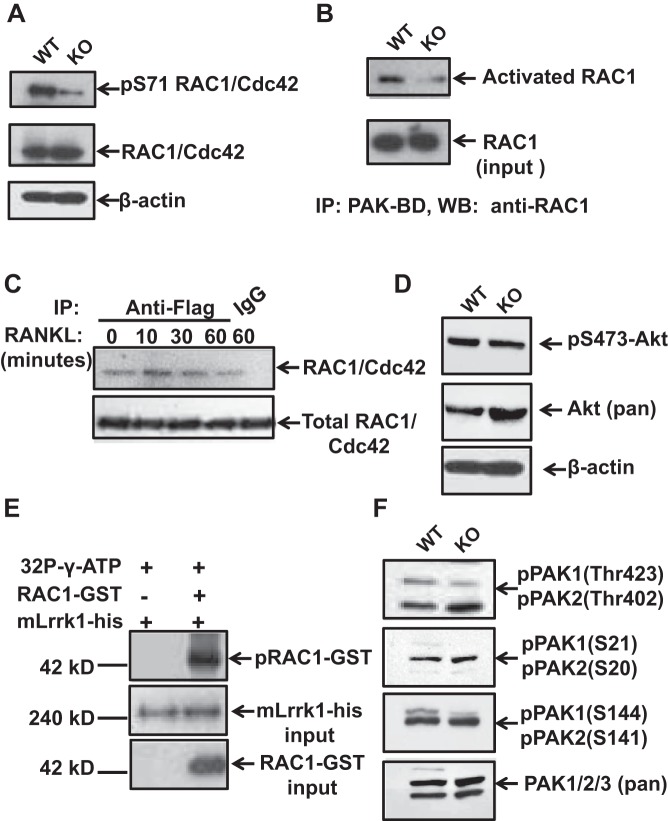
Phosphorylation and activation of small GTPase RAC1/Cdc42 proteins are reduced in Lrrk1-deficient osteoclasts. *A*: Western blot analyses show reduced phosphorylation of RAC1/Cdc42 at Ser^71^. *B*: Pull-down assays show reduced RAC1 binding to p21 protein-activated kinase binding domain (PAK-BD). *C*: RAC1 interacts with hLrrk1 in Raw 264.7 cells in response to RANKL treatment in a time-dependent manner, detected by coimmunoprecipitation. *D*: Western blot analyses show comparable expression of Akt phosphorylation at Ser^473^ in WT and Lrrk1 KO osteoclasts. *E*: RAC1-GST was phosphorylated by recombinant mLrrk1 expressed in vitro. *F*: Western blot analyses show reduced phosphorylation of PAK1 at Ser^144^ and Thr^423^ in Lrrk1 KO osteoclasts compared with WT cells.

On the basis of the findings that Rac1/2 double-KO mice are osteopetrotic and that Rac1 interacted with Lrrk2, a homolog of Lrrk1, we examined the phosphorylation status and activation of small GTPase RAC1/Cdc42 in WT and *Lrrk1* KO osteoclasts. We found that phosphorylation of Rac1/Cdc42 at Ser^71^ was dramatically reduced in Lrrk1-deficient osteoclasts. Total Rac1/Cdc42 protein levels were not changed between WT and KO osteoclasts (Fig. 4*A*). Pull-down assays revealed that Rac1 binding to the binding domain of PAK1 was also reduced in Lrrk1-deficient cells (Fig. 4*B*). To determine whether the Rac1/Cdc42 complex binds to Lrrk1, we performed immunoprecipitation assays in Lrrk1-overexpressing Raw 264.7 cells. We found that, in response to RANKL treatment, Rac1/Cdc42 binding to Lrrk1 increased within 10 min (Fig. 4*C*). Because Akt is known to phosphorylate Rac1 in vitro, we also performed Western blot analyses using an antibody specific to pSer^473^-Akt, an active form of Akt. We found the pSer^473^-Akt level in Lrrk1 KO osteoclasts was not altered compared with the WT cells (Fig. 4*D*). To further determine whether Lrrk1 regulates osteoclast function via phosphorylation and activation of small GTPase Rac1/Cdc42 proteins, we performed an in vitro kinase assay using recombinant mLrrk1-his protein purified from 293T cells and purified Rac1-GST as a substrate. We found that mLrrk1 phosphorylated Rac1-GST in vitro (Fig. 4*E*).

We next determined if the reduced RAC1/Cdc42 activation affects downstream PAK1 phosphorylation and activation in differentiated Lrrk1 KO osteoclasts. Total cellular lysate isolated from mature osteoclasts was analyzed by Western blot using antibodies specific to phosphorylated PAK at serine or threonine residues. We observed that autophosphorylation of PAK1 at Ser^144^, catalyzed by binding to RAC1/Cdc42, was greatly diminished in Lrrk1-deficient multinuclear cells, whereas the total levels of PAK1/2/3 proteins were comparable between WT and Lrrk1 KO cells (Fig. 4*F*). Phosphorylation of PAK1 at Thr^423^, which induces PAK1 activation, was also slightly reduced in Lrrk1-deficient cells.

#### Overexpression of Q61L partially rescues bone-resorptive function of Lrrk1-null osteoclasts.

**Fig. 5.**
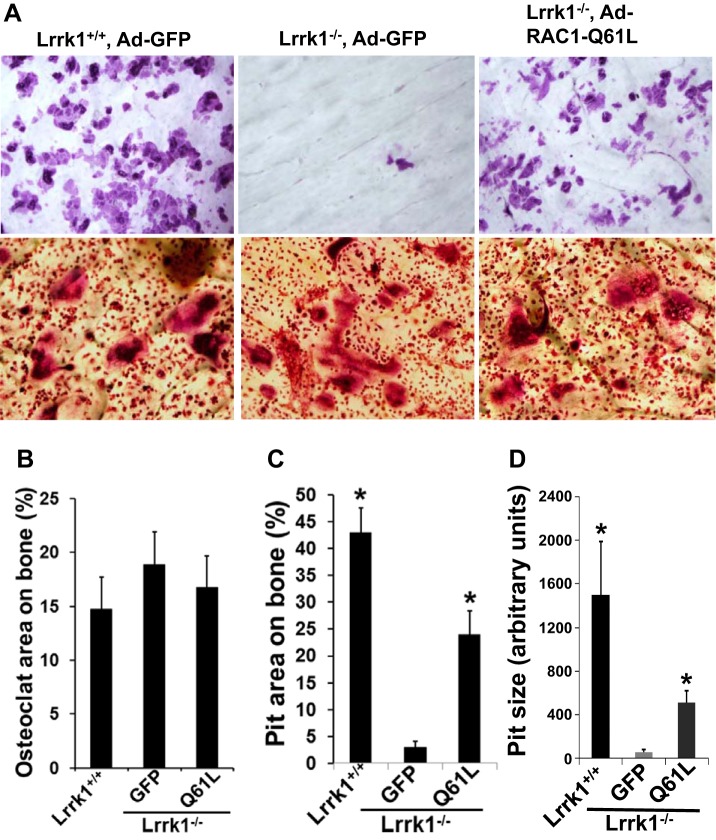
Overexpression of a constitutively active from of RAC1 (Q61L) in *Lrrk1* KO osteoclasts partially rescues bone resorptive function. *A*: representative images of resorptive pits and TRAP staining-positive osteoclasts on bone slices. *B–D*: quantitative data of multinucleated cells, pit area, and pit size on bone slices. *Statistical significance vs. osteoclasts expressing GFP (*P* < 0.01, *n* = 6).

To determine whether expression of a constitutively active form of RAC1 (Q61L) in Lrrk1-deficient osteoclasts can rescue the bone-resorptive defects, precursors derived from *Lrrk1* KO mice were infected Ad-GFP or Ad-Q61L-RAC1 and differentiated into multinuclear cells followed by TRAP staining and pit assays. As shown in Fig. 5, *A* and *B*, the areas of TRAP-positive osteoclasts on bone slices were comparable for WT osteoclasts and *Lrrk1*-deficient cells expressing either GFP or Q61L RAC1 mutant protein. However, there were few pits formed on bone slides in the Lrrk1-deficient cells expressing GFP, while 43 and 24% of bone surfaces were covered with resorptive pits in the WT osteoclasts and Lrrk1-deficient osteoclasts expressing the active form of Q61L RAC1 protein, respectively (Fig. 5*C*). Most of the resorption pits generated by GFP-expressing Lrrk1-deficient osteoclasts remained tiny and shallow compared with the pits obtained from either WT cells or Lrrk1-deficient cells expressing Q61L RAC1 (Fig. 5, *A* and *D*). The pit size in Q61L RAC1-expressing osteoclast cultures, however, was smaller (∼30%) compared with WT osteoclast cultures (Fig. 5*D*).

## DISCUSSION

Previous findings of the dramatic reduction in bone resorption and the persistent resistance to ovariectomy-induced bone loss in the Lrrk1 KO mice (36) and the recent identification of an osteosclerotic patient with an autosomal recessive mutation of Lrrk1 (15) provide unequivocal evidence that Lrrk1 plays a critical role in regulation of bone homeostasis. Mice with disruption of Lrrk1 exhibited severe osteopetrosis phenotypes in the femurs, tibias, and vertebrae due to dysfunction of mature osteoclasts. In contrast to dramatically elevated trabecular bone volume in long bones and vertebra, deficiency of *Lrrk1* had only a mild effect on the skull and mandible. These skeletal phenotypes of *Lrrk1* KO mice were very similar to the clinical phenotypes observed in a patient with a mutant Lrrk1, in which a partial deletion of Lrrk1 resulted in a frame-shift that disrupted the 7th WD40 repeat and added a unique 66-amino acid sequence at the COOH terminus of the Lrrk1 protein. This patient suffered from an osteosclerotic metaphyseal dysplasia, a unique form of osteopetrosis characterized by severe osteosclerosis localized to the metaphyses of the long and short tubular bones. The vertebral bodies and pelvis showed marginal sclerosis while the patient's skull seemed normal, as measured by X-ray radiography (15). The modest skull and mandible phenotypes of *Lrrk1* KO mice and the patient with Lrrk1 mutation compared with the phenotypes of long bones and vertebrae are consistent with evidence that regulation of osteoclast function is different in intramembranous vs. endochondral bone.

The large multidomains of Lrrk1 could function as an adaptor, a kinase, or a GTPase-modulating protein of focal adhesion molecules in osteoclasts. Previous studies have linked mutations in the *Lrrk2* but not *Lrrk1* gene in humans to Parkinson disease, although both Lrrk1 and Lrrk2 are expressed in multiple tissues including macrophage precursors (4, 23, 29, 30, 39). Loss of Lrrk1 in mice caused severe bone phenotypes while mice with disruption of Lrrk2 did not exhibit an obvious skeletal abnormality, indicating that Lrrk1 has a unique function in bone cells that Lrrk2 cannot compensate; thus, the domains which distinguish Lrrk1 from Lrrk2 must determine its specific function in osteoclasts. While both Lrrk1 and Lrrk2 have several common functional motifs, including LRR, ROC, COR, kinase, and WD40 domains, Lrrk1 but not Lrrk2 contains NH_2_-terminal ANK repeats (9, 23). Sequence analysis found that hLrrk1 or mLrrk1 contains four ANK repeats that are highly identical to the ANK repeat consensus sequence. The predicted 3-D structure of hLrrk1 ANK contains functional motifs of β-hairpins, inner helices, and outer helices that can bind to important proteins involved in podosome assembly and disassembly in osteoclasts (5, 11, 19). Interestingly, the integrin-linked serine/threonie kinase (ILK) also contains four ANK repeats that bind to the LIM1 domain of PINCH isoforms and the serine/threonine phosphatase ILK associated phosphatase at the NH_2_ terminus, modulating ILK protein conformation, cellular localization, F-actin remodeling, and integrin signaling (10, 33, 34). Inactivation of the ILK in osteoclasts in mice resulted in an increase in osteoclastogenesis both in vitro and in vivo, but ILK-deficient osteoclasts displayed a decrease in bone resorption (10). Our studies involving deletion mutants of Lrrk1 reveal that bone resorptive defects were rescued by overexpression of mLrrk1 or ΔLRR-mLrrk1 in *Lrrk1* KO osteoclasts. However, overexpression of ΔANK-mLrrk1 failed to rescue the bone resorption function of Lrrk1 KO osteoclasts. Our data are consistent with the prediction that the ANK domain is required for Lrrk1 regulation of osteoclast function.

In addition to the findings that overexpression of the K651A point mutation of mLrrk1 in Lrrk1-null cells could not rescue pit formation (20, 21), our studies also revealed that WD40 repeats are also important for Lrrk1 function in osteoclasts. Although the function of the Lrrk2 WD40 domain has been well studied, little is known on the function of a putative Lrrk1 WD40. It has been reported that the WD40 domain of Lrrk2 can bind and sequester synaptic vesicles via interaction with vesicle-associated proteins, and a G2385R point mutation in the WD40 domain correlated with a reduced binding affinity of Lrrk2 WD40 to synaptic vesicles (25). Molecular modeling also suggests that the G2385 residue is located on the outer surface of the WD40 domain toward the COOH terminus, and the substitution of the neutral and flexible glycine for a passively changed arginine at this position could interfere with interdomain interaction, causing 50% reduction in Lrrk2 kinase activity. Consistent with this study, Jorgensen et al. (16) have shown that hLrrk2 normally exists in a dimeric complex, and deletion of the WD40 domain prevents Lrrk2 dimerization and autophosphorylation. While Lrrk1 also contains a putative WD40 domain, the sequence is quite divergent from the Lrrk2 WD40 domain, suggesting that the two family members may catalyze different biological substrates and interact with distinct protein partners. In our study, we found that overexpression of ΔWD-mLrrk1 did not rescue the bone resorption function of Lrrk1 KO osteoclasts. On the basis of the function of the WD-40 domain of Lrrk2 and human genetic studies on Lrrk1, we assume that the WD-40 domain in Lrrk1 is also required for Lrrk1 dimerization or intermolecular or intramolecular interaction for kinase activation (15, 16). Mutation or deletion of the WD-40 domain may alter kinase conformation leading to inactivation or disruption of protein-substrate interactions or subcellular localization. Future studies on Lrrk1 protein crystallography, domain pull-down assays, and mass spectrometry-based proteomics will confirm these predictions. Our future studies will also focus on the functions of WD40 as well as other functional domains in Lrrk1 in regulating Src phosphorylation and Rac activation.

Lrrk1 contains a PKC-like serine/threonine protein kinase domain, and GTP binding to the Roc domain stimulates Lrrk1 kinase activity in vitro (20, 21). Although we have previously shown that Lrrk1 plays a critical role in regulating osteoclast sealing zone formation, osteoclast activity, and bone resorption due to altered Tyr^527^ phosphorylation of c-Src (36), the molecular pathways underlying Lrrk1 regulation of these functions and the direct substrates are still unknown. Here, we demonstrated that PKC substrates with molecular masses of ∼110, 57–78, and 27 kDa were phosphorylated at significantly lower levels in *Lrrk1* KO osteoclasts compared with WT cells. While several signaling pathways including integrin, NF-κB, and Src could be involved in regulating osteoclast function (14), we focused on small GTPase Rac/Cdc42 signaling pathways since previous studies showed that double KO of Rac1 and Rac2 in mice caused osteopetrosis due to cytoskeleton disarrangement and osteoclast dysfunction (8, 38). While Rac1/2-deficient osteoclasts in vitro and *Rac1/2* KO mice exhibited bone resorption defects, the magnitude of phenotypic changes caused by lack of Rac1/2 is less severe than that of Lrrk1-deficient cells or *Lrrk1 KO* mice. Furthermore, mice with deletion of Rac1/2 in mature osteoclasts also had a normal response to PTH treatment, just as in the case of Lrrk1 KO mice (27, 38). In addition, RAC1 has been reported to interact with Lrrk2 protein (6). These studies strongly suggest that small GTPase Rac1/Cdc42 may be direct biological substrates of Lrrk1. Accordingly, our Western blot and pull-down assays revealed that Lrrk1 deficiency in osteoclasts resulted in reduced phosphorylation and activation of RAC1/Cdc42. In vitro kinase assays confirmed that mLrrk1 phosphorylated RAC1-GST, and immunoprecipitation analyses found that the interaction of mLrrk1 with RAC1 occurred within 10 min after RANKL treatment. Overexpression of constitutively active Q61L RAC1 partially rescued the resorptive function of Lrrk1-deficient osteoclasts. Furthermore, we observed that lack of Lrrk1 in osteoclasts led to reduced PAK1 autophosphorylation, catalyzed by RAC1/Cdc42 binding and activation. On the basis of our findings and previously published data, we conclude that Lrrk1 may regulate osteoclast function via modulation of phosphorylation and activation of small GTPase RAC1/Cdc42 proteins and RAC1/Cdc42 proteins may be direct biological substrates of Lrrk1 in osteoclasts.

Recent studies seem to suggest that there is an extensive cross-talk between integrins, Src, and Rho signaling pathways (14). Our data indicate that osteopetrosis phenotypes in Lrrk1 knockout mice are more severe than the Src knockout, integrin-β3 knockout or RAC1/2 double-knockout mice, although these mice display partially overlapping skeletal abnormalities that are caused by defective bone remodeling and dysfunctional osteoclasts. Lrrk1 seems to modulate signaling pathways that are triggered by various transmembrane receptors and augment the cellular response to a number of growth factors including EGF, M-CSF, and RANKL. Although integrin, Src, and Rac1 are predicted to be localized in the same signaling pathway, these molecules are also regulated by multiple upstream growth factors, protein kinases, and protein phosphatases. Inactivation of the single Src/Rac1 pathway by Lrrk1 deficiency could not explain the severe phenotypes observed in Lrrk1 knockout mice. It is therefore possible that Lrrk1 modulates other signaling pathways besides c-Src and Rac1/Cdc42. Further studies are needed to determine whether phosphorylation or activation of Rac1/Cdc42 is rescued by expression of c-Src or vice versa in Lrrk1-null osteoclasts.

## GRANTS

This research was supported by National Institutes of Health Grant AR066831-01 and ASBMR GAP grant to W. Xing. The funder had no role in study design, data collection and analysis, decision to publish, or manuscript preparation. S. M's salary was supported by a senior research career scientist award from the Department of Veteran's Affairs. The research work was performed at facilities provided by the Department of Veterans Affairs.

## DISCLOSURES

No conflicts of interest, financial or otherwise, are declared by the author(s).

## AUTHOR CONTRIBUTIONS

C.Z., H.G., X.Q., B.L., and W.X. performed experiments; C.Z., H.G., and W.X. analyzed data; C.Z. and W.X. prepared figures; S.M. and W.X. conception and design of research; S.M. and W.X. interpreted results of experiments; S.M. and W.X. edited and revised manuscript; W.X. drafted manuscript; W.X. approved final version of manuscript.
